# Binding Studies of a New Water-Soluble Iron(III) Schiff Base Complex to DNA Using Multispectroscopic Methods

**DOI:** 10.1155/2012/126451

**Published:** 2012-07-31

**Authors:** Nahid Shahabadi, Zeinab Ghasemian, Saba Hadidi

**Affiliations:** Department of Inorganic Chemistry, Faculty of Chemistry, Razi University, Kermanshah 74155, Iran

## Abstract

A novel iron(III) complex [Fe(SF)](ClO_4_)_3_.2H_2_O; in which SF = N,N_0_-bis{5-[(triphenylphosphonium chloride)-methyl] salicylidene}-o-phenylenediamine) has been synthesized and characterized using different physicochemical methods. The binding of this complex with calf thymus (CT) DNA was investigated by circular dichroism, absorption studies, emission spectroscopy, voltammetric studies, and viscosity measurements. The results showed that this complex can bind to DNA via external and groove binding modes.

## 1. Introduction

 Schiff bases, characterized by the azomethine group (–RC= N–), form a significant class of compounds in medicinal and pharmaceutical chemistry and are known to have biological applications due to their antibacterial [1–6], antifungal [3–6], and antitumor [7, 8] activity. Schiff base ligands are considered “privileged ligands” because they are easily prepared by the condensation between aldehydes and imines. The incorporation of transition metals into these compounds leads to the enhancement of their biological activities and decrease in the cytotoxicity of both metal ion and Schiff base ligand [9–11]. Schiff bases with donors (N, O, S, etc.) have structural similarities with neutral biological systems and due to presence of imine group are utilized in elucidating the mechanism of transformation of racemization reaction in biological system [12–14]. Those play an important role in inorganic chemistry as they easily form stable complexes with most transition metal ions.

The structural diversity of transition metal complexes of Schiff base ligands and the structure function relationships of the resulting complexes have been the focus of extensive research in recent years [15–21]. Metal complexes have been widely applied in clinics for centuries, although their molecular mechanism has not yet been entirely understood [22, 23]. The binding and reaction of metal complexes with DNA have been the subjects of intense investigation in relation to the development of new reagents for biotechnology and medicine [24–30]. To understand such functions and to design new DNA-binding metal complexes, investigations of the DNA-binding structures of the complexes are inevitably necessary. Investigations on the interaction between transition metal complexes and DNA has attracted many interests due to their importance in cancer therapy and molecular biology [31–39].

**Figure 1:**
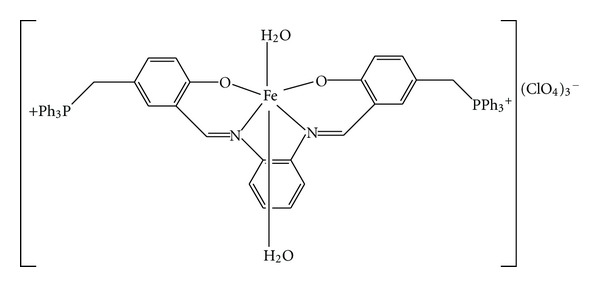
The molecular structure of iron(III) complex.

It is well known that some drugs have higher activity when administered as metal complexes than as free ligands [40]. Among them less explored iron-based Schiff base complexes possess many advantages over traditional catalyst due to iron's copious, nontoxic, and inexpensive nature [41]. In this context, we studied the interaction of a new iron(III) complex of a Schiff base ligand (Figure 1) with calf thymus DNA using physicochemical methods. 

## 2. Experimental

### 2.1. Materials

 O-phenylenediamine, salicylaldehyde, p-formaldehyde, triphenylphosphine, absolute ethanol, iron nitrate nona hydrate, diethyl ether, dichloromethane, ethylacetate, NaClO_4_·H_2_O, EDTA, Tris-HCl, NaOH, potassium acetate, and chloroform, were purchased from Merck. Doubly distilled deionized water was used throughout. Highly polymerized CT-DNA and Tris-HCl buffer were purchased from Sigma. 

Experiments were carried out in Tris-HCl buffer at pH 7.2. Solutions of CT-DNA gave a UV absorbance ratio (260 over 280 nm) of more than 1.8, indicating that the DNA was sufficiently free of protein. The stock solution of CT-DNA was prepared by dissolving approximately 1-2 mg of CT-DNA fibers in 2 mL Tris-HCl (10 mM) by shaking gently and stored for 24 h at 4°C. The DNA concentration (monomer units) of the stock solution (1 × 10^−2^ M per nucleotide) was determined by UV spectrophotometry in properly diluted samples using a molar absorption coefficient of 6600 M^−1^ cm^−1^ at 258 nm. DNA solutions were used after no more than 4 days. 

### 2.2. Instrumentation

 The elemental analysis was performed using a Heraeus CHN elemental analyzer. Absorbance spectra were recorded using an HP spectrophotometer (Agilent 8453) equipped with a thermostated bath (Huber polysat cc1). Absorption titration experiments were conducted by keeping the concentration of complex constant (5 × 10^−5^ M) while varying the DNA concentration from 0 to 2.5 × 10^−4^ M (ri = [DNA]/[complex] = 0.0, 0.15, 0.35, 0.4, 0.55, 0.65, and 0.7). Absorbance values were recorded after each successive addition of DNA solution, followed by an equilibration period. CD measurements were recorded on a JASCO (J-810) spectropolarimeter, keeping the concentration of DNA constant (8 × 10^−5^ M) while varying the complex concentration (ri = [complex]/[DNA] = ri = 0, 0.2, 0.4, 0.6, 0.8, and 1). Viscosity measurements were made using a viscosimeter (SCHOT AVS 450) maintained at 25.0 ± 0.5°C using a constant temperature bath. The DNA concentration was fixed at 5 × 10^−5^ M, and flow time was measured with a digital stopwatch. The mean values of three measurements were used to evaluate the viscosity *η* of the samples. The values for relative specific viscosity (*η*/*η*_0_)^1/3^, where *η*_0_ and *η* are the specific viscosity contributions of DNA in the absence (*η*_0_) and in the presence of the complex (*η*), were plotted against ri (ri = [DNA]/[complex] = 0.0, 0.2, 0.4, 0.6, 0.8, and 1.0). All fluorescence measurements were carried out with a JASCO spectrofluorimeter (FP6200) by keeping the concentration of complex constant while varying the DNA concentration from 0 to 5 × 10^−5^ M (ri = [DNA]/[complex] = 0.0, 0.08, 0.16, 0.32, 0.4, 0.48, 0.55, 0.62, 1, and 1.1) at three different temperatures (298, 310, and 288 K). The cyclic voltammetric (CV) measurements were performed using an *μ*-AUTOLAB model (PG STAT C), with a three-electrode system: a 0.10 cm diameter Glassy carbon (GC) disc as working electrode, an Ag/AgCl electrode as reference electrode, and a Pt wire as counter electrode. Electrochemical experiments were carried out in a 25 mL voltammetric cell at room temperature. All potentials are referred to the Ag/AgCl reference. Their surfaces were freshly polished with 0.05 mm alumina prior to each experiment and were rinsed using double distilled water between each polishing step. The supporting electrolyte was 0.05 M of Tris-HCl buffer solution (pH 7.4) which was prepared with double distilled water. The current-potential curves and experimental data were recorded on software NOVA 1.6. 

### 2.3. Synthesis of the Iron(III) Schiff Base Complex

 To a vigorously stirred solution (10 mL) of 3-formyl-4 hydroxybenzyl-triphenyl phosphonium chloride (0.12 g, 2 mmol) was first added Fe(NO_3_)_3_·9H_2_O (0.136 g, 2 mmol) dissolved in water (l0 mL) and then Et_3_N (pH 7.5–8). A few minutes later, an ethanolic solution (2 mL) of o-phenylenediamine (0.015 g, 1 mmol) was added drop wise. The solution turned to dark brown and the mixture was vigorously stirred and refluxed for 3 h under an argon atmosphere. After that, NaClO_4_·H_2_O (2 mmol) was dissolved in a minimum amount of water (5 mL) and added to the reaction mixture. The resulting brown powder precipitate was collected by filtration, washed with cold ethanol and ether, and dried in air. The complex was dissolved in CH_2_Cl_2_ (6 mL), Ethylacetate (3 mL) was added and the resulting solution was allowed to evaporate slowly at room temperature. The solid product was filtered off and washed with ethyl acetate and ether yielding the iron complex as a brown powder. 

## 3. Results and Discussion

### 3.1. Synthesis and Characterization of Iron(III) Complex

 The iron(III) complex of SF ligand (SF = N, N_0_-bis{5-[(triphenylphosphonium chloride)-methyl] salicylidene}-o-phenylenediamine) synthesized by mixing 3-formyl-4 hydroxybenzyl-triphenyl phosphonium chloride, Fe(III) nitrate and o-phenylenediamine in high yield. FT-IR spectrum of the complex was recorded in KBr pellets from 4000 to 400 cm^−1^. The FT-IR spectrum of the Schiff base was characterized by the appearance of a band at 3424 cm^−1^ due to the (OH) group [42]. In the FT-IR spectrum of the iron(III) complex, the absence of this band indicates deprotonation of the donor and involvement oxygen in bonding with the metal atom. However, the strong band at ca. 1627 cm^−1^ assigned to (CN) of the Schiff base ligand [43] which shifts approximately 16 cm^−1^ to the lower wave numbers upon coordination to metal [42]. Since the iron(III) is paramagnetic in nature, its NMR spectrum could not be obtained. Elemental analysis also confirms the synthesis of the complex. FeC_58_H_52_N_2_P_2_O_16_Cl_3_ anal. calc.: C, 55.39; H, 4.13; N, 2.22. Found: C, 55.21; H, 4.30; N, 2.45.

The electronic spectrum of the complex shows the band at 260 nm is attributable to intramolecular *π* → π* transition. Another band at 340 nm could be attributed to metal to ligand charge transfer. The other weaker bands are attributed to *d* → *d* transitions of metal center. Molar conductance of the complex in DMF is 289 *Ω* ^−1^ cm^2^ mol^−1^ that is indicative of 3 : 1 electrolytic nature of the complex. Conclusive evidence of the bonding was also shown by the observation that new bands in the FT-IR spectra of the metal complex appearing in the low frequency region at 420–518 cm^−1^ characteristic to -(M–O) and -(M–N) stretching vibrations respectively that were not observed in the spectrum of free ligand.

### 3.2. Viscosity Measurements

**Figure 2:**
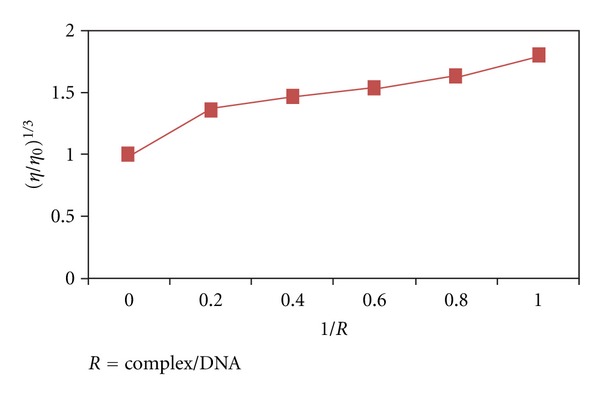
Effect of increasing amounts of iron complex on the viscosity of calf thymus DNA (5 ×10^−5^ M) in 10 mM Tris HCl buffer (ri = 0.2, 0.4, 0.6, 0.8, and 1).

 Hydrodynamic methods that are sensitive to length are regarded as one of the least ambiguous and most critical tests of a binding mode in solution in the absence of crystallographic structural data. Intercalating agents are expected to elongate the double helix to accommodate the ligands in between the base leading to an increase in the viscosity of DNA. In contrast, complex that bind exclusively in the DNA grooves by partial and/or non-classical intercalation, under the same conditions, typically cause less pronounced (positive or negative) or no change in DNA solution viscosity [44]. The values of (*η*/*η*_0_)^1/3^ were plotted against [complex]/[DNA] (Figure 2). With the increase in the amount of the complex, the relative viscosity of DNA increases steadily which is consistent with DNA groove binding which is also known to enhance DNA viscosity [42]. 

As intercalation causes a significant increase in viscosity of DNA solutions due to lengthening of the DNA helix as base pairs are separated to accommodate the aromatic chromophore of the bound molecule, it is tempting to ascribe the observed increase in viscosity to intercalative interaction of the complex. However, the effective intercalation of the phenyl rings of SF in iron complex is discouraged by several steric factors. This type of steric clash has been also suggested for the binding of [Cu(phen)_2_]^+^ and [Ru(phen)_3_]^2+^ complexes, where intercalation of a phen ligand is inhibited by steric interactions involving the other phen ligands with DNA surface. Therefore, it is obvious that the complex prefers to engage in DNA surface binding with its large size affecting an increase in DNA viscosity, rather than in intercalative DNA interaction. The slow increase in viscosity is an indication of groove binding [45, 46].

### 3.3. CD Spectral Studies

**Figure 3:**
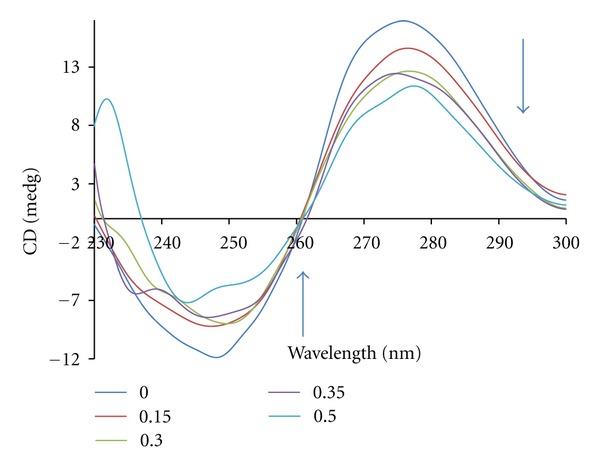


 Circular dichroic spectral techniques give us useful information on how the conformation of DNA is influenced by the binding of the metal complex to DNA. The observed CD spectrum of calf-thymus DNA consists of a positive band at 277 nm due to base stacking and a negative band at 245 nm due to helicity, which is characteristic of DNA in the right-handed B form. While groove binding and electrostatic interaction of small molecules with DNA show little or no perturbations on the base stacking and helicity bands, intercalation enhances the intensities of both the bands, stabilizing the right-handed B conformation of CT-DNA. The interaction between the complex and CT-DNA was studied by CD spectroscopy (Figure 3).

In this case, the intensities of both the negative and positive bands decrease significantly (shifting to zero levels). This suggests that the DNA binding of the complex induces certain conformational changes, such as the conversion from a more B-like to a more C-like structure within the DNA molecule [47]. These changes are indicative of a non-intercalative mode of binding of the complex and offer support to its groove binding nature [48].

### 3.4. Fluorescence Spectroscopy

**Figure 4:**
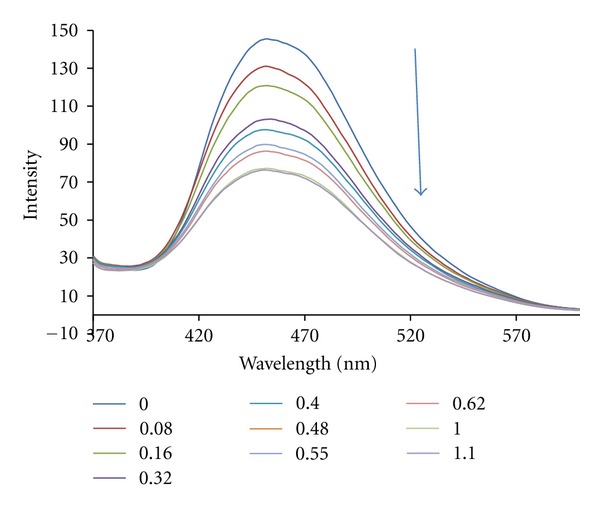


 Quenching can occur by different mechanisms which are usually classified as dynamic and static quenching. Dynamic quenching refers to a process in which the fluorophore and the quencher come into contact during the transient existence of the exited state, while static quenching refers to fluorophore-quencher complex formation. In general, dynamic and static quenching can be distinguished by their differing dependence on temperature and excited-state lifetime. Since in both cases the fluorescence intensity is related to the concentration of the quencher, the quenched fluorophore can serve as an indicator for the quenching agent [49]. The effect of DNA on the fluorescence intensity of the complex is shown in Figure 4. Upon addition of CT-DNA, a decrease in emission intensity was observed for the complex. This implies that the title complex has a strong interaction with DNA. The quenching of the luminescence of the complex by CT-DNA may be attributed to the photoelectron transfer from the guanine bases of DNA to the excited MLCT state, as reported for other complexes [50, 51].

 Fluorescence quenching is described by the Stern-Volmer equation:

(1) F0F=1+Kqτ0[Q]=1+Ksv[Q],

where *F*_0_ and *F* represent the fluorescence intensities in the absence and in the presence of quencher, respectively. *Kq* is the fluorophore quenching rate constant, *K*_sv_ is quenching constant,*τ*_0_ is the lifetime of the fluorophore in the absence of a quencher (*τ*_0_ = 10^−8^), and [Q] is the concentration of quencher [52]. Dynamic and static quenching can be distinguished by their different dependence on temperature [53]. The results in Table 1 indicate that the probable quenching mechanism of this complex by CT-DNA involves static quenching, because *K*_sv_ decreases with increasing temperature [54].

#### 3.4.1. Binding Constant and the Number of Binding Sites

 The binding constant (*K*_*f*_) and the binding stoichiometry (*n*) for the complex formation between iron complex and DNA were measured using (2) [55]

(2) Log(F0−FF)=LogKf+nLog[Q].

Here, *F*_0_ and *F* are the fluorescence intensities of the fluorophore in the absence and presence of different concentrations of CT-DNA, respectively. The values of *K*_*f*_ aaa and *n* were found to be 3.54 × 10^4^ M^−1^ and 0.64, respectively, (Table 2).

#### 3.4.2. Binding Mode between the Complex and DNA

**Figure 5:**
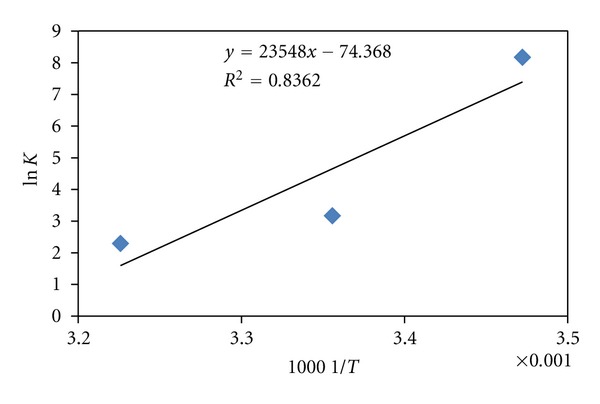


 According to the thermodynamic data, interpreted as follows, the model of interaction between a drug and biomolecule can be [56]: (1)  Δ*H *> 0 and Δ*S *> 0, hydrophobic forces; (2)  Δ*H *< 0 and Δ*S *< 0, van der Waals interactions and hydrogen bonds; (3)  Δ*H *< 0 and Δ*S *> 0, electrostatic interactions [57]. In order to elucidate the interaction of our complex with DNA, the thermodynamic parameters were calculated. The plot of ln*K* versus 1/*T* (Figure 5; (3)) allows the determination of Δ*H* and Δ*S*. If the temperature does not vary significantly, the enthalpy change can be regarded as a constant. Based on the binding constants at different temperatures, the free energy changes can be estimated (Table 3; (4)) by the following equations

(3) LnK=−ΔHRT+ΔSR,

(4) ΔG=ΔH−TΔS=−RTLnK,

where *K* is the Stern-Volmer quenching constant at the corresponding temperatures and R is the gas constant. When we apply this analysis to the binding of the complex with CT-DNA, we find that Δ*H *< 0 and Δ*S *< 0. Therefore, van der Waals interactions or hydrogen bonds are probably the main forces in the binding of the titled complex to CT-DNA.

### 3.5. Absorption Studies

**Figure 6:**
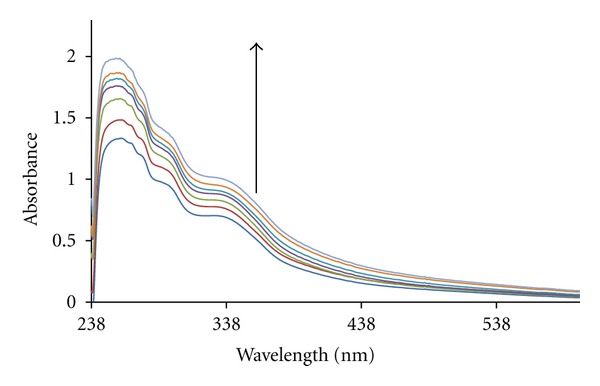


 Electronic absorption spectroscopy was an effective method to examine the binding mode of DNA with metal complexes. In general, hypochromism and red shift are associated with the binding of the complex to the helix by an intercalative mode involving strong stacking interaction of the aromatic chromophore of the complex between the DNA base pairs.  Figure 6 shows the UV-absorption spectra of the complex in the absence and presence of DNA. In the ultraviolet region from 200 to 400 nm, the complex had strong absorption peaks at 260 nm, besides a shoulder band around 340 nm. The absorption intensity of the complex sample increased (hyperchromism; %H = 37.3%) and no red shift evidently after the addition of DNA, which indicated the interactions between DNA and the complex. The hyperchromism of the complex, on addition of calf-thymus DNA, implies that the binding mode is non-intercalative in nature. This hyperchromism can be attributed to external contact (surface binding) with the duplex. Some similar hyperchromism have been observed [58–60]. But this needs further clarification of the DNA binding mode of the complex by viscosity measurements.

The absorption data were analyzed to evaluate the intrinsic binding constant, *K*_*b*_, which can be determined from (5) [61],

(5) [DNA](εa−εf)=[DNA](ε0−εf)+1Kb(εb−εf),

where [DNA] is the concentration of DNA in base pairs, the apparent absorption coefficient **ε*a*, and **ε*f* and **ε*b* correspond to A*obsd*/[M], the extinction coefficient of the free compound and the extinction coefficient of the compound when fully bound to DNA, respectively.

**Figure 7:**
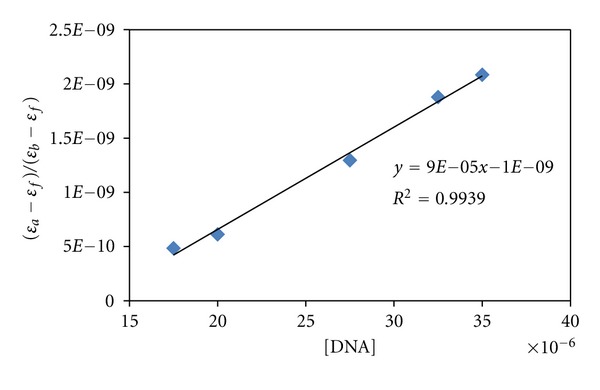


In plots of [DNA]/(**ε*a* − **ε*f*) versus [DNA], *K*_*b*_ is given by the ratio of slope to the intercept. The intrinsic bindingconstant, *K*_*b*_, of complex was 9 × 10^4^ M^−1^ (Figure 7).

The *K*_*b*_ value obtained here is lower than that reported for classical intercalators (for ethidium bromide and [Ru(phen)DPPZ] whose binding constants have been found to be in the order of 10^6^-10^7^ M) [62, 63]. The observed binding constant is more in keeping with the groove binding with DNA, as observed in the literature [64]. 

The *K*_*b*_ value for the complex is comparable to that observed for some complexes like, [Co (phen)_3_]^3+^ (1.6 × 10^4^ M^−1^) [65]; [Ru (phen)_3_)^2+^] (0.55 × 10^4^ M^−1^) [66]; (Ru (phen)_2_(bpy)](PF_6_)_2_ (0.36 × 10^4^ M^−1^) [67]; [Ru (phen)_2_L]^+4^ (2.5 × 10^4^ M^−1^) [68] (electrostatic binding mode); [Ru (tpy)(pph_3_)_2_Cl]^+^ (*K*_*b*_ = 4.1 × 10^4^ M^−1^, *s* = 0.34) [69]; [Ru (tpy)(Asph_3_)Cl]^+^ (*K*_*b*_ = 4.5 × 10^4^ M^−1^, *s* = 0.1) [69]; tricationic Co(III) complexes with asymmetric ligand, [Co(phen)_2_(pdta)]^3+^ (*K*_*b*_ = 2.8 × 10^4^ M^−1^) [70]; [Co (bpy)_2_(CNOIP)]^3+^ (*K*_*b*_ = 5 × 10^4^ M^−1^) [71].

### 3.6. Voltammetric Studies

The application of electrochemical methods to the study of metallointercalation and coordination of transitional metal complexes to DNA provides a useful complement to the previously used methods of investigation, such as UV–vis spectroscopy [72, 73].

**Figure 8:**
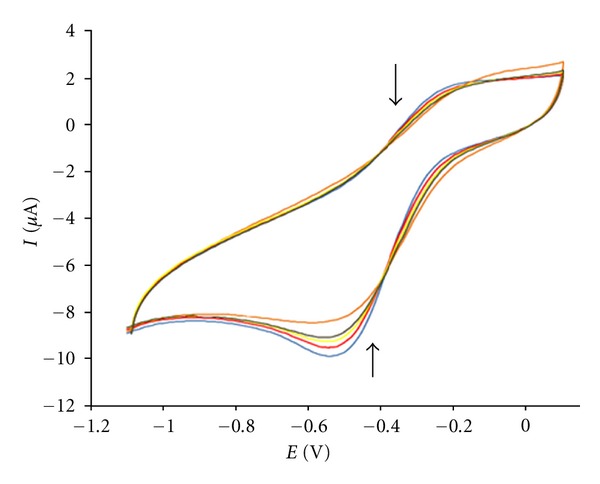


The electrochemical behavior of complex is well known, and was strongly influenced by the electrode material. A well-defined and sensitive peak was observed from the solution of the complex with a GC electrode rather than the Pt one. Therefore, a GC electrode was used in this investigation. When CT-DNA is added to a solution of the complex both the anodic and cathodic peak current heights of the complex decreased in the same manner of increasing additions of DNA, (Figure 8).

The substantial diminution in peak current is attributed to the formation of slowly diffusing complex-DNA supramolecular complex due to which the concentration of the free drug (mainly responsible for the transfer of current) is lowered [74].

The gradual decay in peak current of the complex by increasing of DNA concentration, ranging from 20–100 *μ*M, can be used to quantify the binding constant by the application of the following equation [75]:

(6) log⁡(1[DNA])=log⁡K+log⁡(II0−I),

where *K* is the binding constant, *I*_0_ and *I* are the peak currents of the free guest and the complex, respectively. 

**Figure 9:**
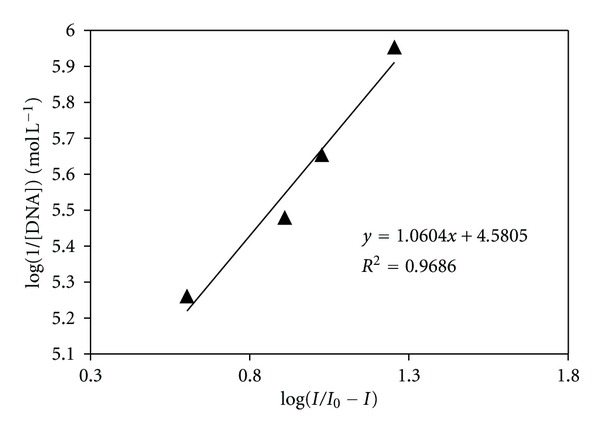
Cyclic voltammogram of 0.1 mM Fe(III) complex on a polished glassy carbon electrode in the absence and presence of different concentrations of DNA. v: 100 mVs^−1^, buffer: 0.05 M Tris-HCl buffer (pH 7.4).

The binding constant with a value of 3.81 × 10^4^ mol^−1^ L was obtained from the intercept of log⁡(1/[DNA]) versus log⁡(*I*/(*I*_0_ − *I*)) plot (Figure 9).

The values of binding constants obtained from UV-vis absorption, fluorescence spectroscopy and CV measurements, 9 × 10^4^, 3.54 × 10^4^ and 3.81 × 10^4^ mol^−1^ L, respectively, were in close agreement.

From the results of the above experiments, the formation of an electrochemically nonactive complex of the complex with DNA resulted in the decrease of the free concentration of the complex in the reaction solution, which caused the decrease of the peak current.

## 4. Conclusion

 One of the most important goals of pharmacological research is the search for new molecular structures which exhibit effective antitumor activities. This has driven inorganic and organometallic chemists to look for new metal compounds with good activities, preferably against tumors that are responsible for high-cancer mortality. In this study, binding interaction of a water soluble iron(III) complex of a Schiff base, SF, (SF 1/4 N, N_0_-bis{5-[(triphenylphosphonium chloride)-methyl] salicylidene}-o-phenylenediamine) with calf thymus DNA has been investigated and the results show that the complex can bind to DNA via groove binding mode. 

## Figures and Tables

**Table 1:** The quenching constants of the Fe(III) complex by CT-DNA at different temperatures.

Temperature (*K*)	*R* ^2^	*K*_SV_ (L mol^−1^) × 10^4^	*K*_*q*_ (L mol^−1^) × 10^12^
288	0.992	7.046	7.046
298	0.971	6.276	6.276
310	0.921	2.683	2.683

**Table 2:** Binding constants (*K*_*f*_) and the number of binding sites (*n*) of the complex DNA system.

Temperature (*K*)	Linear equation	*R* ^2^	*n*	*K*_*f*_	Log*K*_*f*_
288	*Y* = 1.678*X* + 8.176	0.990	1.678	1.49 × 10^8^	8.176
298	*Y* = 0.644*X* + 4.549	0.987	0.644	3.54 × 10^4^	4.549
310	*Y* = 0.527*X* + 2.293	0.957	0.527	1.96 × 10^2^	2.293

**Table 3:** Thermodynamic parameters and binding constants for the binding of Fe(III) complex to calf thymus DNA.

Temperature (*K*)	Δ*G* (KJ mol^−1^)	Δ*H* (KJ mol^−1^)	Δ*S* (J mol^−1 ^K^−1^)
283	−21.99	−197.45	−620.05
299	−12.07	−197.45	−620.05
310	−5.25	−197.45	−620.05
